# Uncovering the novel characteristics of Asian honey bee, *Apis cerana*, by whole genome sequencing

**DOI:** 10.1186/1471-2164-16-1

**Published:** 2015-01-02

**Authors:** Doori Park, Je Won Jung, Beom-Soon Choi, Murukarthick Jayakodi, Jeongsoo Lee, Jongsung Lim, Yeisoo Yu, Yong-Soo Choi, Myeong-Lyeol Lee, Yoonseong Park, Ik-Young Choi, Tae-Jin Yang, Owain R Edwards, Gyoungju Nah, Hyung Wook Kwon

**Affiliations:** Biomodulation Major, Department of Agricultural Biotechnology and Research Institute of Agriculture and Life Sciences, College of Agriculture and Life Sciences, Seoul National University, Seoul, 151-921 Republic of Korea; National Instrumentation Center for Environmental Management, College of Agriculture and Life Sciences, Seoul National University, Seoul, 151-742 Republic of Korea; Department of Plant Science, College of Agriculture and Life Sciences, Seoul National University, Seoul, 151-921 Republic of Korea; Arizona Genomics Institute, University of Arizona, Tucson, Arizona 85721 USA; National Institute of Agricultural Biotechnology, Rural development Administration, Suwon, 441-707 Republic of Korea; Department of Entomology, Kansas State University, Manhattan, Kansas USA; CSIRO Ecosystem Sciences, Centre for Environment and Life Sciences, Underwood Avenue, Floreat, WA 6014 Australia

**Keywords:** *Apis cerana*, Asian honey bee, Genome, Social insect, Chemosensory receptors, Honey bee immunity

## Abstract

**Background:**

The honey bee is an important model system for increasing understanding of molecular and neural mechanisms underlying social behaviors relevant to the agricultural industry and basic science. The western honey bee, *Apis mellifera*, has served as a model species, and its genome sequence has been published. In contrast, the genome of the Asian honey bee, *Apis cerana*, has not yet been sequenced. *A. cerana* has been raised in Asian countries for thousands of years and has brought considerable economic benefits to the apicultural industry. *A cerana* has divergent biological traits compared to *A. mellifera* and it has played a key role in maintaining biodiversity in eastern and southern Asia. Here we report the first whole genome sequence of *A. cerana*.

**Results:**

Using *de novo* assembly methods, we produced a 238 Mbp draft of the *A. cerana* genome and generated 10,651 genes. *A.cerana*-specific genes were analyzed to better understand the novel characteristics of this honey bee species. Seventy-two percent of the *A. cerana*-specific genes had more than one GO term, and 1,696 enzymes were categorized into 125 pathways. Genes involved in chemoreception and immunity were carefully identified and compared to those from other sequenced insect models. These included 10 gustatory receptors, 119 odorant receptors, 10 ionotropic receptors, and 160 immune-related genes.

**Conclusions:**

This first report of the whole genome sequence of *A. cerana* provides resources for comparative sociogenomics, especially in the field of social insect communication. These important tools will contribute to a better understanding of the complex behaviors and natural biology of the Asian honey bee and to anticipate its future evolutionary trajectory.

**Electronic supplementary material:**

The online version of this article (doi:10.1186/1471-2164-16-1) contains supplementary material, which is available to authorized users.

## Background

The honey bee, a social insect, has received considerable attention as a model for studying neurobiology [1], development [2], social behavior [3] and, most recently, epigenomics [4]. To facilitate studies of this important biological model, the genomic sequence of the western honey bee, *Apis mellifera*, was published in 2006, providing a wealth of information for understanding the molecular basis of social behavior and eusocial evolution [5].

Honey bees are classified in the order Hymenoptera, which also includes ants, bees, sawflies, and wasps [6]. The genus *Apis* consists of eight Asian species and one western species [7]. Ancient bees first emerged 120–130 million years ago (mya) coincident with the emergence of early angiosperms [8]. The ancestors of modern bees were geographically isolated in the Middle East during the late Pleistocene approximately 1–2 mya. It was from this population that the ancestral lineage of *A. mellifera* spread to Africa, and the ancestral lineages of *A. cerana* expanded throughout Europe and Asia [7, 9, 10]. As a result of allopatric speciation, these two honey bee species may have evolved in different ecological environments, which gave rise to different behavioral and physiological characteristics observed in the present day [7].

Compared to *A. mellifera*, the Asian honey bee, *A. cerana*, has several distinguishing behavioral traits (Table 1). It is able to adapt to extreme weather conditions [11] and has long flight duration [7], effective grooming and hygienic behaviors [12], and cooperative group-level defenses [13]. A well-known behavior of *A. cerana* is aggregation when a colony is exposed to dangers, such as predators or intruders. In these situations, guard bees produce alarm pheromones that dictate group behavior [13–15]. In addition, *A. cerana* provides considerable economic benefits to the apicultural industry through its high quality bi-products, perhaps even more so than *A. mellifera*[16].

**Table 1:** **Comparison of biological differences between** ***A. cerana*** **and** ***A. mellifera***

	***A. cerana***	***A. mellifera***	Reference
Aria of origin	Southeast Asia	Africa or Western Asia	
Body mass	43.8 mm	77.2 mm	[17]
Wing length (worker)	7.54 ± 1.14 mm	9.32 ± 0.16 mm	[18]
Wing beat frequencies (worker)	306 beats/s	235 beats/s	
Nest cavity volume	10-15 liters in general	35 liters,	[19]
Comb structure	Uneven round	Square	
Dismantling of old comb	Yes	No	[4]
Pore in the drone cell	Yes	No	[20]
Flight pattern	Rapid, hasty, and unpredictably zig-zag	Steady and clumsy	[4]
Homing speed (~50 m)	192 sec	295 sec	[21]
Foraging range	Maximum 1500 m to 2500 m	Average 1650 m, maximum 6 km	[22]
Active foraging time and temperature range	9 am and 11.30 am/15.5 - 21°C	11 am and 1 pm/21–25°C	[23]
Flower preference	Wide range of flowers, including wild plants	Mainly on *Trifolium* and *Brasscia*	[24]
Pollination	Larger pollen collector	Smaller pollen collector (compared to *A. cerana*)	[10]
Robbing defense	Weak	Strong	[10]
Frequencies of absconding	Often (heavily depends on condition)	Rarely (heavily depends on condition)	[8]
Collecting propolis	No	Yes	[4]
Defense to wasps	Forming a ball with worker	Individual	[11, 12]
*Varroa* mite resistance	Yes	No	[13]
Ventilation direction	Head toward outside	Head toward entrance	[7, 12]
Royal jelly production rate	3.21 ± 0.43 g	80.5 ± 7.83 g	[23]
Rate of stinging	Low	High	[4]

In recent years, population decreases similar to those documented in the western honey bee have also been seen in *A. cerana* colonies [25]. In Korea, more than ninety percent of *A. cerana* colonies were destroyed by sacbrood virus (SBV) infection [26]. However, few studies have been conducted on the underlying molecular mechanisms and immune responses to this virus [11].

This study reports the *A. cerana* genome produced with deep sequence coverage using next-generation technologies. We generated gene sets of *A. cerana* using transcriptome data from seven tissues. Then, we focused on the characterization of important genes related to chemosensory reception and immunity. This genome sequence will provide invaluable information on the novel characteristics of the honey bee species indigenous to eastern and Southern Asia. The data will also provide a resource for comparative sociogenomic studies with the seven ants and western honey bee species for which genomes are already available [5, 27–30].

## Results and discussion

### Genomic features of *A. cerana*

#### Sequencing and assembly

We performed whole genome sequencing of the Asian honey bee using seven drones derived from a single colony. Because the honey bee has a haplodiploid mating system, males (drones) are haploid and females (workers and queens) are diploid. To minimize possible contamination from foreign genomes such as bacteria and viruses, we eliminated mid-gut tissues from the individual drone bees prior to sequencing. Genomic sequence libraries were constructed with a combination of short reads (500 bp) and two longer insert libraries (3 and 10 Kb), using Illumina sequencing technology (152-fold coverage) (Table 2). The assembly consisted of 2,430 scaffolds with a total length of 228 Mb, which covered 96% of the estimated genome size (238 Mb) [31]. General information on the genome assembly is presented in Table 3. The N50 scaffold size was 1,421 kb (Table 3), much longer than the N50 scaffold size found in the initial and recently improved assemblies of *A. mellifera* (359 kb and 997 kb, Amel_4.0 and Amel_4.5, respectively; Additional file 1: Table S1) [32]. To assess the accuracy of the scaffolds, we compared the genome of *A. mellifera* and *A. cerana* to identify genomic synteny (Additional file 1: Figure S1). Results revealed several scaffolds of *A. cerana* and chromosome 3 of *A. mellifera* that showed syntenic relationships with no large-scale rearrangement. Additionally, we found that the mitochondrial genome of *A. mellifera* (NCBI GQ162109, [33]) and one contig of *A. cerana* had high sequence similarity, ~99% (Additional file 1: Figure S2). This contig, covering the entire *A. cerana* mitochondrial genome, is 15,915 bp and includes 13 protein-coding genes (Additional file 1: Figure S3). All sequence information was submitted in the NCBI [Submission number: SUB582139].

**Table 2:** **Sequencing raw data summary**

Library	Raw data		Filtered data		Sequence coverage (X)
	Number of reads	Length (bp)	Number of reads	Length (bp)	
PE^a^ 500 bp	290,571,627	58,695,468,654	101,647,459	20,532,786,718	86
MP^b^ 3 kb	214,359,129	21,435,912,900	87,974,983	8,797,498,300	37
MP 10 kb	229,499,284	22,949,928,400	68,408,294	6,840,829,400	29
Total	734,430,040	103,081,309,954	258,030,736	36,171,114,418	152

The raw data were filtered by high stringency and more detailed information described in Method. ^a^Paired end sequencing, ^b^Mate-paired sequencing.

**Table 3:** **Genome assembly summary**

Summary	Number
Estimated genome size (bp)	238,934,385
Total assembly length (bp)	228,315,917
Total assembly gap length (bp)	22,358,847
Number of contigs	18,160
Contig N50 length (bp)	21,729
Number of scaffolds	2,430
Scaffold N50 length (bp)	1,421,626
Largest scaffold length (bp)	6,352,280
Average scaffod length (bp)	93,957
Number of (A + T)s (%)	60.19
Number of (G + C)s (%)	30.02
Number of Ns (%)	9.79
Repeats length (bp)	14,794,603
Interspersed repeats (bp)	4,448,613
Simple repeats (bp)	8,167,274

Size of estimated genome and statistics of assembled scaffolds. The N50 scaffod size indicated that 50% of nucleotides in the assembly occur in scaffolds of length more than or equal to the N50 size.

#### Guanine plus cytosine (GC) content

The *A. cerana* assembly contains 30% GC (Table 3), similar to the mean GC content of *A. mellifera* (33%). In addition, six ant species (*Linepithema humile*, *Camponotus floridanus*, *Pogonomyrmex barbatus*, *Solenopsis invicta*, *Atta cephalotes*, and *Acromyrmex echinatior*) have similar GC contents ranging from 33% to 38% [5, 27–30, 34, 35]. In contrast, *Drosophila melanogaster* (42%), *Nasonia vitripennis* (42%), and *Harpegnathos saltator* (45%) have higher GC contents compared to *A. cerana*[34, 36]. According to comparative studies of two ant species, *C. floridanus and H. saltator*, organisms with more complex social traits may have AT-biased genomes [30, 34].

**Figure 1:**
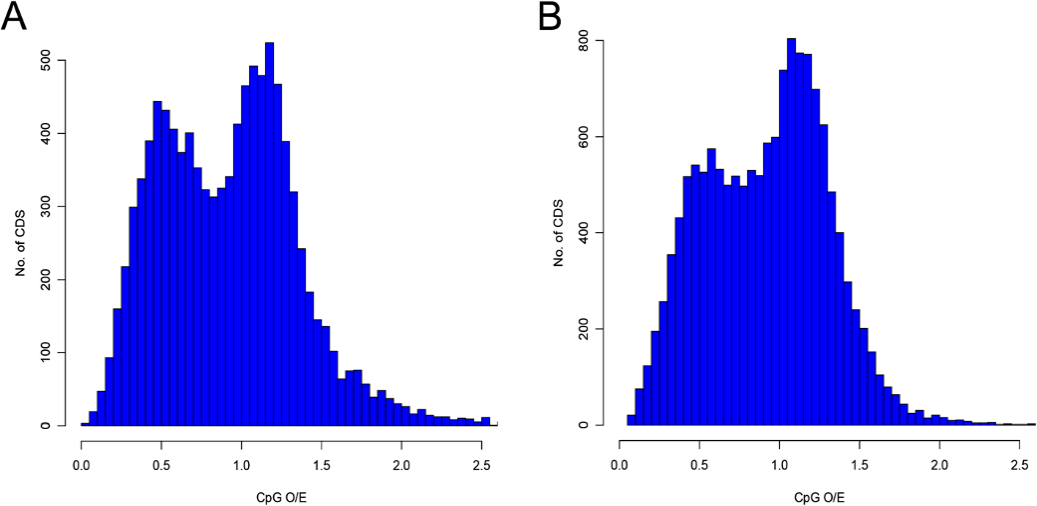
**CpG analysis of protein sequence of** ***A. mellifera*** **and** ***A. cerana.*** Distributions of normalized CpG o/e content of the **(A)** *A. cerana* and **(B)** *A. mellifera*. Bimodal distributions of honey bee protein-coding sequences indicate that the genome of honey bee encoded two distinct classes of genes which are targeted by DNA methylation.

Relative AT bias correlates with DNA methylation, as DNA methyltransferases (*Dmnts*) are almost entirely targeted to cytosine residues followed by guanines in the 5′ to 3′ orientation (CpG dinucleotides). Methylcytosine tends to mutate to thymine (T), thus the gradational accumulation of mutation that convert CpG dinucleotides to TpG dinucleotides lead to AT-rich genomes [37–39]. In particular, normalized CpG observed/expected (CpG o/e) values have a negative relationship with levels of DNA methylation [40]. DNA methylation is one of the major part of epigenetic regulation and has functional roles in gene expression regulation in vertebrates and insects [40]. In contrast to vertebrate genomes, which are depleted of CpG dinucleotides [41], most hymenopteran insects, including *A. cerana* (1.61), *A. mellifera* (1.65), *C. floridanus* (1.58), *H. saltator* (1.49), and *N. vitripennis* (1.35), exhibit high levels of CpG o/e in their genomes [34]. Another intriguing discovery is that normalized CpG o/e value within protein coding sequences of *A. cerana* showed bimodal distribution, similar to *A. mellifera* (Figure 1, Additional file 1: Figure S4) and the pea aphid *Acyrthosiphon pisum*[5, 42]. Interestingly, two distinct classes of genes are documented to perform different functions [43], which low-CpG genes are mainly involved in housekeeping function and high-CpG genes are involved in development. Indeed, we found that genes that are represented low-CpG classes are categorized with metabolic process, and transcriptional and translational regulation. In contrast, high-CpG genes represented GO categories that specific to biological functions.

The genome of *A. cerana*, *A. mellifera*, and *A. pisum* encode full complements of DNA methylation proteins (*Dmnts*) [5, 42], but, according to a recent discovery, several insects possess a full set of *Dnmts* without any striking depletion pattern of coding exons [27, 36, 44]. Thus, this genomic feature may not be species specific, but mechanisms of epigenetic regulation may be conserved in both honey bee species.

#### Repetitive elements

The *A. cerana* assembly comprised 6.48% (14.79 Mb) repetitive elements, consisting of 3.58% (8.16 Mb) simple repeats and 1.95% (4.44 Mb) interspersed repeats (Additional file 1: Table S2). Seventy-five *A. cerana*-specific repeat elements were found using the *de novo* repeat finding program, RepeatModeler (version 1.0.7). Since the *A. cerana* genome assembly contained 9.79% N’s, we assumed that repetitive sequences in the current assembly may be underestimated. In comparison to *A. mellifera*, only long terminal repeat elements were over-represented in *A. cerana*, which accounted for 0.1% (218 kb) of the genome, compared to 0.02% (49.6 kb) in *A. mellifera*. In contrast, long interspersed elements and short interspersed elements were not detected in the genome of *A. cerana*. Both were found in the *A. mellifera* genome at frequencies of 0.04% (83.1 kb) and 0.03% (70 kb), respectively. DNA transposons constitute 0.11% (247 kb) of the *A. cerana* genome and 0.57% (1.34 Mb) of the *A. mellifera* genome. *Mariner* transposable elements, first discovered in the fruit fly, have been found across honey bee species [45, 46]. The genome of the western honey bee, *A. mellifera*, contained multiple copies of *mariner* transposons, ranging from *AmMar1* to *AmMar6*[5]. In contrast, the *A. cerana* genome contained orthologs of *AmMar1* and *AmMar3*-*6*, but *AmMar2* ortholog was not found. This is consistent with the speculation that *AmMar1* and *AmMar2* were transferred to the *A. mellifera* genome relatively recently [45, 46].

Although genome-wide repeat analyses need to be investigated further, the results of this study showed a striking reduction of transposable elements (TEs) and retrotransposons in the *A. cerana* genome compared to *A. mellifera*. Lack of TEs is one of the major features of the honey bee genome, compared to other sequenced Hymenoptera [30, 32]. Some evidence suggests that grooming and hygienic behaviors in eusocial organisms reduced insertion of TEs from foreign genomes [30]. However, both social and non-social hymenopteran insect genomes, including seven ants and the parasitoid *Nasonia*, have been sequenced, and they include significantly different quantities of repetitive elements comprising 11% to 28% of genomes [30, 36]. Therefore, hygienic behavior is not the only factor influencing the accumulation of repetitive elements in genomes.

### Analysis of the *A. cerana*gene set

Due to limited data on expressed sequence tags (ESTs) and complementary DNAs (cDNAs) available for *A. cerana*, we established a gene annotation pipeline using multiple evidence data (Table 4). First, we generated 213,327 transcripts covering 515,809,639 bp using a *de novo* assembly from 68 Gb of *A. cerana* RNA-seq reads. Second, RNA-seq data was aligned to scaffold sequences, which resulted in 31,027 gene models representing 96,495,948 bp. Third, we performed computational gene prediction based on the scaffold sequence information, which generated 24,579 genes covering 18,397,306 bp. We also employed *A. mellifera* gene sequences collected from the National Center for Biotechnology Information Reference Sequence Database (NCBI RefSeq, [47]) as a model to obtain homology-based gene annotation. Subsequently, we merged all predicted gene models using the MAKER [48] program to generate a primary gene set. All genes were queried with the NCBI non-redundant database using BLASTX. Lastly, we manually checked for missing genes, partial genes, or separated genes. Chemoreceptor genes, including gustatory receptors (Grs), odorant receptors (Ors), and ionotropic receptors (Irs), were investigated more carefully using analyses of functional sequence domains. Finally, 10,651 genes were annotated as the official gene set (OGS) of *A. cerana*, OGS version 1.0 (Table 4), of which approximately 84% of genes were annotated with NCBI non-redundant database and 70% were annotated in the Uniprot database [49]. Overall, the total number of genes in the *A. cerana* OGS v1.0 was comparable to the number in the *A. mellifera* OGS v1.0 (10,157 genes). However, the number is less than the current release of the *A. mellifera* genome, OGS v3.2 (15,314 genes; Table 5) [5, 32].

**Table 4:** **General statistics for gene modeling**

	Number of gene models	Length (bp)
**Initial evaluation (Evidence data)**		
RNA-seq, *de novo*	213,327	515,809,639
Genome + RNA-seq	31,027	96,495,948
Genome sequence, *de novo*	24,579	18,397,306
*A. mellifera* RefSeq^a^	18,215	23,654,031
**Primary gene set**		
Integrated evidence data	11,458	32,199,638
**Final gene set (OGS v1.0)**		
Manual annotation	10,651	15,655,993

^a^NCBI RefSeq from http://www.ncbi.nlm.nih.gov/genome/48?project_id=13343.

**Table 5:** **Comparison of the official gene set of** ***A. cerana*** **and** ***A. mellifera***

	***A. cerana*** OGSv1.0 (2014)	***A. mellifera*** OGSv1.0 (2006)	***A. mellifera*** OGSv3.2 (2014)
Number of genes	10,651	10,157	15,314
Total bases (bp)	15,655,993	16,484,776	19,342,383
Minimum length (bp)	33	24	75
Maximum length (bp)	37,824	53,649	70,263
Average length (bp)	1,469	1,623	1,263
Median length (bp)	1,071	1,197	840
(A + T)s (%)	61.88	60.41	60.42
(G + C)s (%)	38.12	39.43	39.47
Ns (%)	0.00	0.16	0.10

We classified genes by function using the gene ontology (GO) and Kyoto Encyclopedia of Genes and Genomes (KEGG) databases [50, 51]; 6,338 genes (60%) had more than one GO term and 1,696 enzymes were categorized into 125 pathways (Additional files 2 and 3). Here, several interesting molecular pathways that could represent honey bee-specific molecular mechanisms were revealed. For example, the fatty acid biosynthesis, glutathione metabolism, and cytochrome P450 pathways may be involved in nest-mate recognition and detoxification of pesticides (Additional file 1: Figure S5) [52, 53]. The surface of the honey bee is composed of fatty acids and hydrocarbons, which reflect identity, and guard bees recognize these compounds to discriminate colony members from intruders [52]. KEGG analyses revealed that classes of enzymes involved in fatty acid biosynthesis are shared between *A. cerana* and *A. mellifera*, and *A. cerana* has fewer detoxification enzymes compared to fly and mosquito but similar numbers to *A. mellifera*[53]. The contribution of pesticides to global colony losses of *A. mellifera* is still a controversial issue, but data indicate that *A. mellifera* is unusually sensitive to various insecticides [54, 55]. Interestingly, colonies of *A. cerana* have not shown similar levels of collapse to *A. mellifera*, but this could be explained by other differences that may reduce exposure to pesticides, such as frequent absconding behavior, small nest architecture, and foraging in high altitude regions [7, 56].

### Genes unique to *A. cerana*and orthologous to honey bee

**Figure 2:**
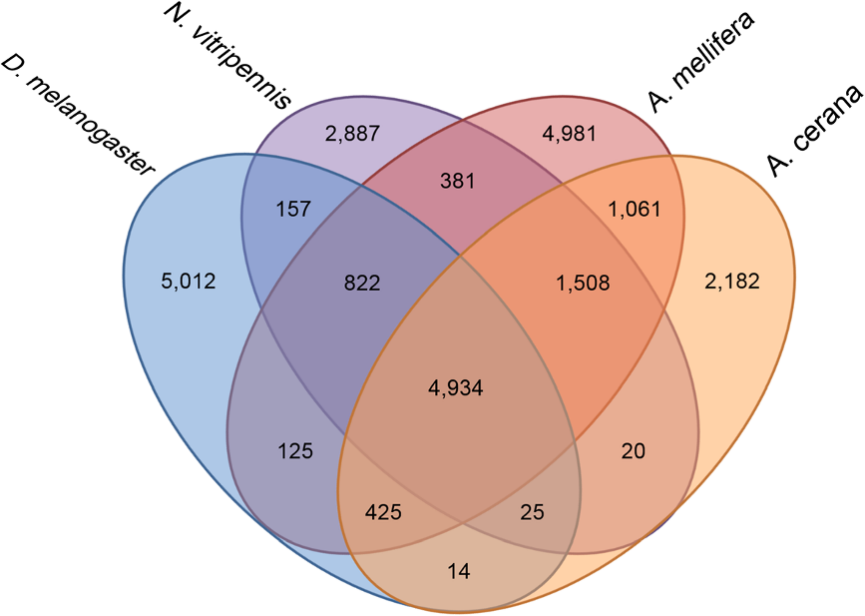
**Comparative analysis of orthologous protein groups among the four insect genomes.** Orthology analysis of the proteins of *A. cerana* (orange oval) with three well known insect models, *D. melanogaster* (blue oval), *N. vitripennis* (purple oval), and *A. mellifera* (red oval). Both *D. melanogaster* and *N. vitripennis* are non-social and *A. mellifera* and *A. cerana* are social insect species. *indicates *A. cerana*-specific proteins.

To investigate whether non-orthologous genes are associated with features of *A. cerana* biology, we compared three hymenopteran insects, *Apis mellifera* (social), *Apis cerana* (social), *Nasonia vitripennis* (non-social), and one dipteran insect, *Drosophila melanogaster* (non-social) by orthology based clustering. Amongst 2,182 unique genes in *A. cerana* (Figure 2), most of the significantly enriched GO-terms were involved in neuromuscular junction, neuromuscular process, regulation of muscle organ development, muscle cell differentiation, and muscle tissue development (*p* < 0.05, Additional file 4). *A. cerana* has a higher wing beat frequency (306 beats/s) compared to *A. mellifera* (235 beats/s) and quick, impetuous, and unpredictable flight patterns, therefore some of the enriched proteins involved in muscle movement might account for *A. cerana*-specific flight patterns [56]. Future studies should be performed to dissect this relationship.

Notably, neural signaling-related GO-terms, including neuron recognition, signaling receptor activity, transmembrane receptor signaling pathway, ionotropic glutamate receptor signaling pathway, and active transmembrane transporter activity, which are closely related with chemosensory reception and chemical signaling, were also enriched (*p* < 0.05) in the *A. cerana* unique gene set (Additional file 4). Genes involved in chemical signaling have evolved rapidly, especially in eusocial organisms [57, 58]. Neural signaling processes play major roles mediating social communication in honey bee society. *A. cerana* shows a number of group-level behaviors distinct from *A. mellifera*[12], including a unique defense behaviors against a hornets. *A. cerana* guard bees raise their abdomens and shake or flutter, producing alarm pheromones, when hornets approach the hive [7, 56]. Additional research is required to determine whether molecular regulation mechanisms found uniquely in *A. cerana* may be responsible for these unique social defense behaviors.

Since *A. mellifera* and *A. cerana* diverged recently, we hypothesized there would be protein orthologs conserved in both honey bee species that explain shared honey bee characteristics. A total of 1,061 *A. cerana* proteins were identified with orthologs in *A. mellifera* but no other non-social species. These orthologs were categorized with GO-terms “sensory perception of smell” (*p* < 1.75E^−04^) and “sensory perception of chemical stimulus” (*p* < 7.55E^−04^), which are crucial features for social communication and physical interaction [27]. In addition, the GO-term “carbohydrate transporter activity” (*p* < 1.87E^−02^), which describes cuticular hydrocarbon detection [27], and “regulation of short-term neuronal synaptic plasticity” (*p* < 2.21E^−02^) and “transmembrane signaling receptor activity” (*p* < 3.04E^−02^), which are involved in neuronal signaling during social interaction, were also enriched in orthologs shared by the two honey bee species (Additional file 1: Table S3).

### Chemoreceptor gene family

Chemoreceptors play important roles in communication and social behaviors, in part by mediating detection of chemical signals from nest-mates [59]. Major groups of chemoreceptor genes include gustatory receptors (*Grs*), odorant receptors (*Ors*), and ionotropic receptors (*Irs*) [59–63]. In social insects, such as ants and honey bees, chemical communication is crucial for colony maintenance and cooperation [64–66]. Here, we have characterized 10 new *Grs*, 119 new *Ors*, and 10 new *Irs* in the *A. cerana* genome. Gene expression patterns, examined using RNA-seq data, revealed that annotated chemoreceptor genes were well organized and comparable to those of *A. mellifera*[59] and *N. vitripennis*[62], although they were slightly underrepresented compared to the *A. mellifera* genome [59].

#### The gustatory receptor family

The gustatory receptor family plays an important role in taste and is used to collect nectar and pollen for energy and brood care [67]. In honey bee society, colony members have division labor and perform different tasks. Nurse bees take care of the brood and the queen, and they clean inside the nest. Forager bees collect food or resin from outside and bring it to the hive [68]. Peripheral and internal regulation of *Gr* gene expression is involved in this behavioral transition [69].

**Figure 3:**
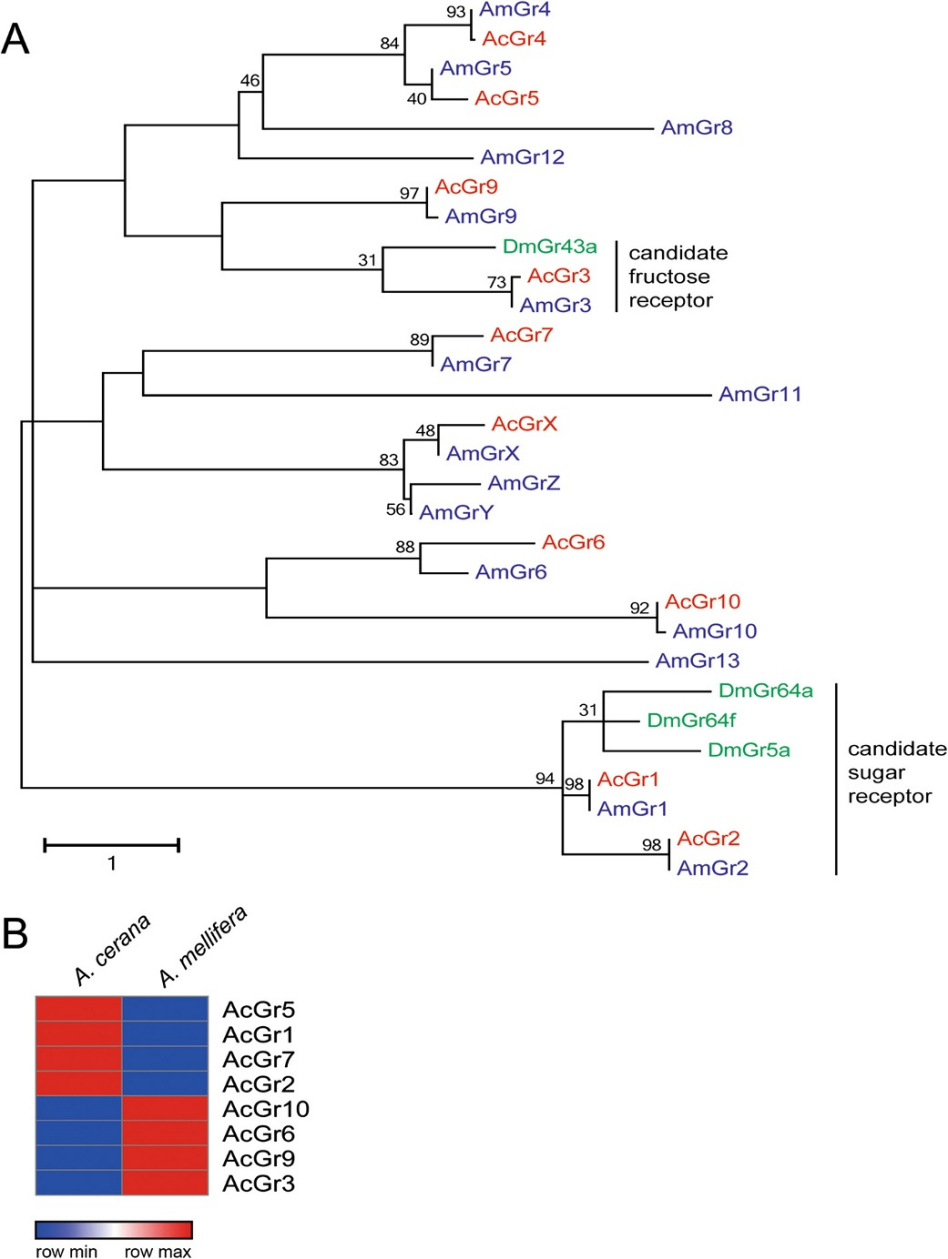
**Phylogenetic tree of the gustatory receptor (** ***Gr*** **) family. (A)** Phylogenetic tree constructed using *A. cerana* (red), *A. mellifera* (blue), and *D. melanogaster* gustatory receptor proteins **(B)** Relative *Gr* gene expression profiling using RPKM values in *A. cerana* (left) and *A. mellfera* (right). Red color indicates high expression compared to blue.

According to Robertson and Wanner [59], the western honey bee, *A. mellifera* has 13 *Grs* (H. M. Robertson, personal communication), a small number compared to the fruit fly *D. melanogaster* (68 *Grs*, [60]), the mosquito *Aedes aegypti* (79 *Grs*, [70]), the parasitoid wasp *N. vitripennis* (58 *Grs*, [62]), and the ant *Linepithema humile* (116 *Grs*, [27]). Similar to *A. mellifera*, 10 *Gr* genes were identified in the *A. cerana* genome. They were named based on their orthology to *A. mellifera Grs* (*AmGrs*). All identified *Grs* in *A. cerana* showed simple orthologous relationships with *Grs* in *A. mellifera*, and *AcGr1*, *2*, *3*, *6*, *7*, *9*, and *10* also had orthologs in *N. vitripennis* (Additional file 1: Figure S6). These data indicated that *Gr* genes are highly conserved among hymenopteran species. Similar to the *A. mellifera Gr* repertoire, *AcGr1* and *AcGr2* were positioned in expanded lineages to sugar receptors of *D. melanogaster*, including *DmGr5a, DmGr61a* and *DmGr64a/f* (Figure 3A) [71–73]. In addition, *AcGr3* shared a clade with *DmGr43a*, which functions as a fructose receptor in the periphery and a nutrient sensor in the brain of *Drosophila* (Figure 3A) [74]. In contrast, *AcGr6*, *7*, *9*, *10,* and *X* lineages showed no apparent relationships with *DmGrs*, implying that they may be unique to the honey bee. Bitter taste receptors also seem to be lost in the *A. cerana* genome, which may be related to the evolution of flower preference in the honey bee [59] compared to other social insects such as the ant in which bitter receptors are preserved [27, 28]. Additionally, orthologs to *Drosophila* carbon dioxide (CO_2_) receptors, *Gr21a* and *Gr63a*[28, 75], were not present in the *A. cerana* genome, similar to *A. mellifera*[59]. However, honey bees are known to detect CO_2_[76], indicating that they may have evolved novel molecular mechanisms similar to the acid sensing mechanism found in *Drosophila* for detecting high CO_2_ concentrations [77]. Partial sequences of *A. cerana Gr4* and *Gr5* orthologs were located using TBLASTN searches. A *Gr8* ortholog could not be found in the *A. cerana* genome.

Expression patterns of *Gr* orthologs in *A. cerana* and *A. mellifera* were determined by relative gene expression analyses (Figure 3B). Surprisingly, expression patterns of *Gr* orthologs between the two honey bee species were distinct. Candidate sugar receptors, *Gr1* and *Gr2*, were expressed higher in *A. cerana* compared to *A. mellifera* (Figure 3B), suggesting that *A. cerana* may have a greater ability to sense sugars. Similarly, *Gr5* and *Gr7* were highly expressed in *A. cerana* compared to *A. mellifera*. In contrast, *Gr3*, *6*, *9*, and *10* were more highly expressed in *A. mellifera* compared to *A. cerana. Gr4* and *GrX* were not detected in antennal transcriptome of *A. cerana* (data not shown), implying that *Gr4* and *GrX* might be expressed at undetectable levels or in other tissues, such as the tongue or legs. Future functional studies on *Grs* may reveal taste sensing and internal regulation differences between the species.

#### The odorant receptor family

Insect odorant receptors play important roles in environmental signal recognition and inter- and intra-species communication [59]. Honey bees use odorant receptors in contexts, including kin recognition, food navigation, and pheromone detection [59, 65, 78]. However, despite the importance of odorant receptors, the functional identification of *Ors* in honey bees is lacking compared to other model insects, including fly and mosquito species [79].

**Figure 4:**
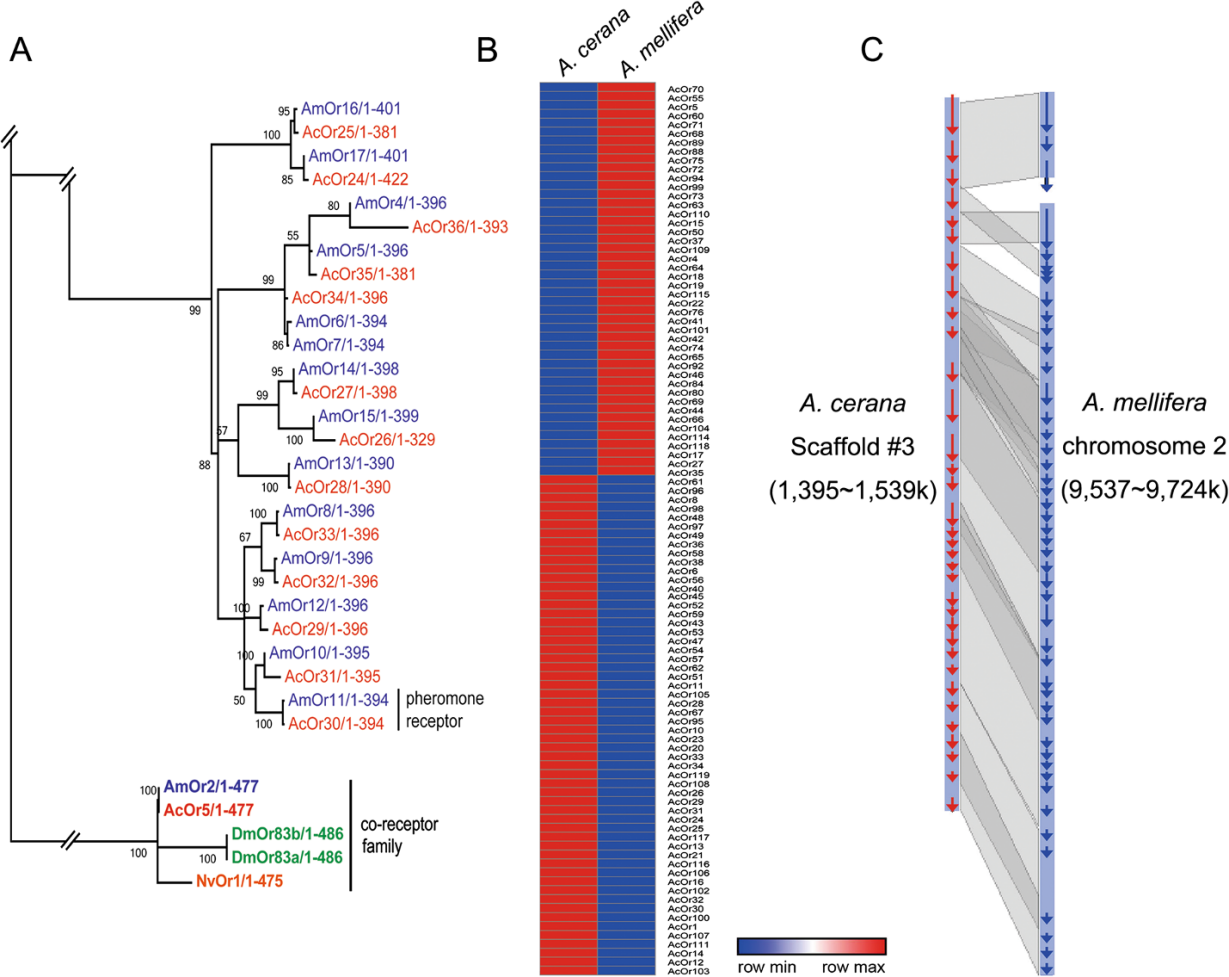
**Phylogenetic tree of the odorant receptor (** ***Or*** **) family. (A)** Phylogenetic tree constructed using *A. cerana* (red), *A. mellifera* (blue), and *D. melanogaster* odorant receptor proteins. **(B)** Relative *Or* gene expression profiling using RPKM values in *A. cerana* (left) and *A. mellfera* (right). Red color indicates high expression compared to blue. **(C)** Microsynteny between *A. cerana* and *A. mellfera Or* genes. Orthologous and paralogous of *A. cerana* (red) and *A. mellifera* (blue) Ors were analyzed with BLASTZ. *A. cerana* scaffold number and *A. mellifera* chromosome number are on the left and right side, respectively.

In the *A. cerana* genome, 119 *AcOrs*, including a few 5′- or 3′- partial sequences containing the odorant receptor domain, were identified. We named *A. cerana Ors* by sequence positions in scaffolds. Most *AcOrs* were not spread evenly across scaffolds, but were clustered at a few locations in the genome. For example, clusters of 37 *Ors*, 15 *Ors*, and 17 *Ors* were arrayed on scaffold 3, 103, and 139, respectively (Additional file 1: Figure S7). In *A. mellifera*, the largest tandem array of 60 *Ors* was found on chromosome 2 [59]. This expansion of *Ors* implies unequal crossing-over by neighboring genes occurred. The large number of *Or* paralogs indicate diverse roles for odorant recognition in honey bee society, such as pheromone blends, cuticular hydrocarbons, and floral odor cocktails [5]. Since *A. mellifera* and *A. cerana* diverged recently [7], it was hypothesized that there may be synteny between *Or* clusters. Regions of *A. mellifera* chromosome 2 with microsynteny conservation were identified by comparing the Or gene arrangement in the *A. cerana* genome to the *A. mellifera* genome. Consistent with the hypothesis, conserved microsynteny and clear orthologs of *A. cerana Or*s to *A. mellifera Or*s were found (Figure 4C, Additional file 5), suggesting that honey bee *Or* paralogs are clustered in conserved genomic regions [80, 81].

Insects have a number of variable *Ors*, which form a chaperone with *olfactory receptor co-receptor* (*Orco*) *in vivo*[82–85]. In the present study, *A. cerana Or5* shared orthology with insect *Orcos* including *D. melanogaster Or83b*, *N. vitripennis Or1*, and *A. mellifera Or2* (Figure 4A). Overall, identified *AcOrs* showed simple orthologous relationships with *AmOrs*, such as 1:1, 1:2, and 1:3 (*AcOrs* : *AmOrs*).

Among 177 *A. mellifera Ors*, *AmOr11* was functionally characterized as a queen pheromone receptor responding to 9-oxo-2-decenoic acid (9-ODA) [78]. In our study, *AcOr30* showed 1:1 orthology to *AmOr11* with 98.7% identity (Additional file 1: Figure S7), implying that queen pheromone components may be conserved between *A. mellifera* and *A. cerana*.

Transcriptome data revealed that *Or* homologs are differentially expressed between *A. cerana* and *A. mellifera* (Figure 4B). Forty-four *Or* homologs were more highly expressed in *A. mellifera*, and 56 *Or* homologs were more highly expressed in *A. cerana*. Different expression patterns support the idea that coding sequences are well conserved among *Or* homologs, but their promoter sequences have diverse regulatory motifs. These data imply that the two honey bee species express different odorant spectra. Specifically, seven *AcOrs* (*AcOr21*, *38*, *40*, *45*, *56*, *58*, and *116*) were expressed only in *A. cerana*, indicating functions specific to *A. cerana*. Functional studies using heterologous expression systems are needed to better understand the various functions of *Ors* in honey bees.

#### The ionotropic receptor family

Recently, a new family of chemosensory receptors, the ionotropic receptor (*Ir*) family, was identified in *D. melanogaster*[61]. Irs in *D. melanogaster* constitute distinct and divergent subfamilies of ionotropic glutamate receptors (*iGluRs*) [63]. Sixty-six *Ir* homologs have been identified in *D. melanogaster*, and 16 were expressed specifically in antennae [61, 63]. This suggested that Irs belong to two subgroups: conserved antennal Irs and species-specific divergent Irs. These subgroups represent classes of Ors and Grs, respectively [63]. In contrast to Ors, which respond broadly to alcohols, ketones, and esters, Irs primarily respond to acids, amines, and carbon dioxide, which can be physiologically important in many insect species [77, 86–88]. Although the functions of these receptors are not yet known, Irs may have more general function in the detection of environmental chemicals including odorants and tastes [89].

**Figure 5:**
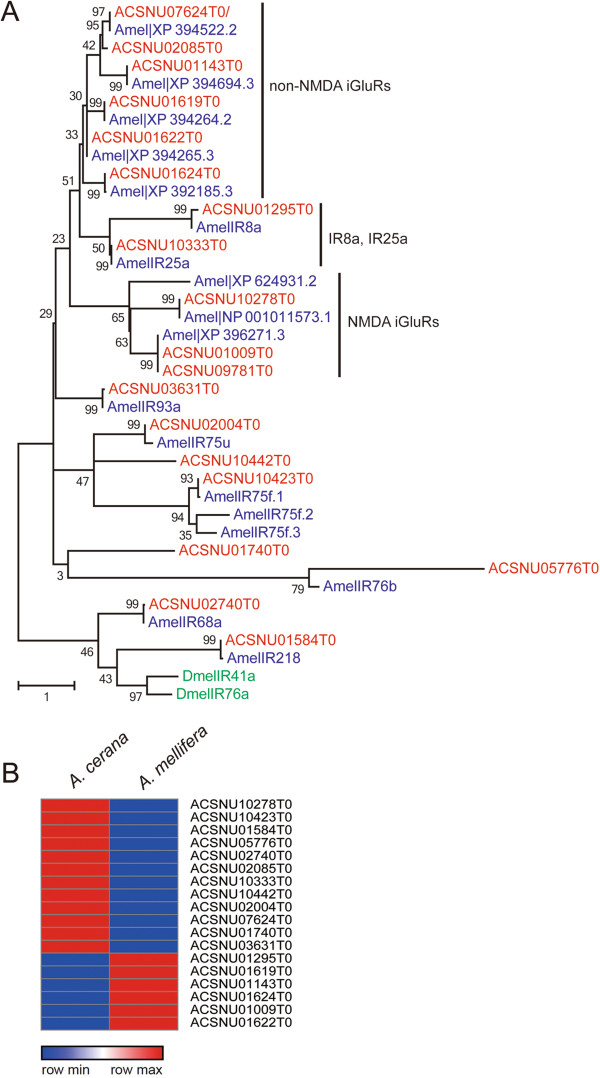
**Phylogenetic tree of the ionotropic receptor (** ***Ir*** **) family. (A)** Phylogenetic tree constructed using *A. cerana* (red), *A. mellifera* (blue), and *D. melanogaster* ionotropic receptor proteins. **(B)** Relative *Ir* gene expression profiling using RPKM values in *A. cerana* (left) and *A. mellfera* (right). Red color indicates high expression compared to blue.

The number of identified *Irs* in insects is increasing [63, 90, 91], and a large complement of *Irs* has been described in the complete genomes of four hymenopteran species: *A. mellifera* (10 *Irs*), *N. vitripennis* (10 *Irs*), *L. humile* (32 *Irs*), and *P. barbatus* (24 *Irs*) [5, 27, 28, 36]. In this study, 10 *Ir* homologs were found in the *A. cerana* genome (Figure 5A). Sequence comparison and phylogenetic analyses of *Irs* with *D. melanogaster* and *A. mellifera* identified putative orthologs of conserved Irs in the *A. cerana* genome: *Ir8a*, *Ir25a*, *Ir68a*, *Ir75a*, *Ir76a*, and *Ir93a*[63]. As expected, highly conserved orthologs of antennal Irs were identified in the *A. cerana* genome. These results support the hypothesis that antennal expression of *Ir* orthologs has been conserved for over 350 mya since dipteran and hymenopteran insects diverged [92]. Other Irs in *A. cerana* with low similarity to orthologs of other insect receptors appear to be honey bee-specific. These Irs may be used for species-specific recognition, including candidates for cuticular hydrocarbon receptors and brood pheromone receptors. However, expression patterns for the vast majority of Irs are unknown and no ligands for honey bee Irs have been identified. In this study, *AcIr* expression profiles were different in *A. mellifera* and *A. cerana* (Figure 5B). Their functions and evolutionary basis for diversity remain to be investigated.

### Immune-related genes

Honey bees are invaluable models for studying social defense dynamics and individual molecular and behavioral defense mechanisms [93]. In contrast to *A. mellifera*, *A. cerana* is not susceptible to the ectoparasitic mite, *Varroa destructor*, one of the major vectors of bee pathogens. In contrast, *A. cerana* has suffered greatly from viral and bacterial diseases in recent years [94]. A recent report indicated that more than 90% of Asian honey bee colonies collapsed due to sacbrood virus (SBV) infection in Korea [95]. Many Asian countries also suffered declines in *A. cerana* colonies for several reasons. However, the molecular defense mechanisms of *A. cerana* are still unknown. Therefore, we investigated immune genes present in the *A. cerana* genome by comparing genomic information to other sequenced insect genomes [96].

**Figure 6:**
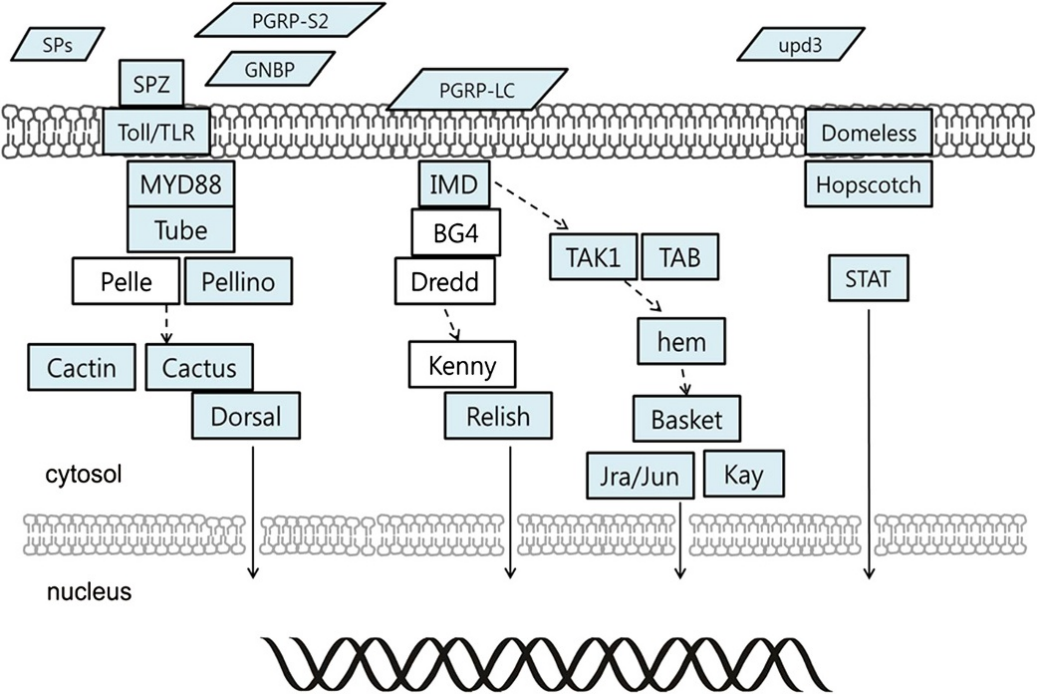
**Candidate genes of immune-related pathways in** ***A. cerana.*** Colored boxes indicate counterparts of immune pathway components in the *A. cerana* genome. Schematic drawing adapted from immune pathways in *A. mellifera*[90].

Using multiple TBLASTN searches, 160 immune gene orthologs were identified in *A. cerana* and 11 additional genes were detected by manual annotation. All major pathways were identified in *A. cerana*, including components of the Toll, Imd, Jak/Stat, and JNK pathways [96]. Notably, the *FADD*, *Dredd*, and *Kenny*, components of the Imd pathway and *Pelle* of the Toll pathway were not detected in the *A. cerana* genome (Figure 6). The total number of innate immune genes in *A. cerana* is similar to other social Hymenoptera [28] (Additional file 1: Table S4), and most immune genes in *A. cerana* shared higher sequence similarity with *A. mellifera* compared to other sequenced insect species. This may be explained by conservation of the innate immune system between *A. cerana* and *A. mellifera*. Eusocial insects have additional social immune systems, such as cleaning behaviors (hygienic behavior, grooming, and undertaking), thermal defenses (*A. mellifera* lacks this behavior), and antibiotic nest architecture (resin collection), which may contribute to reducing exposure to pathogens [93].

Previous studies indicate more antimicrobial proteins are encoded in the *A. cerana* genome compared to *A. mellifera*[11]. Indeed, defensive peptides, including venomous peptides, in *A. cerana* are more strongly expressed than those in *A. mellifera*[97]. In addition, some reports show the uniqueness of strong behavioral defenses of *A. cerana*, such as hygienic and grooming behaviors [11, 12, 94]. Together, these data indicate that *A. cerana*, through a combination of elaborate molecular and behavioral mechanisms, may have a more effective social defense system compared to *A. mellifera*. Functional studies of immune genes will inform knowledge of *A. cerana*-specific disease control methods and provide a valuable model for comparative studies of social insect immune systems.

## Conclusion

The Asian honey bee, *A. cerana*, a close relative of the western honey bee, *A. mellifera*, provides considerable economic benefits to the apicultural industry and serves as a new model for biodiversity in eastern and southern Asia. Despite similarities in Asian and western honey bees, differences in biological traits led us to explore the genome and transcriptome of *A. cerana*. This is the first report to elucidate the whole genome sequence of the Asian honey bee, *A. cerana*, by employing *de novo* assembly of the *A. cerana* genome and computational gene prediction followed by manual annotation. *A. cerana* has unique features for muscle movement and neural signaling, reflecting the wild nature of *A. cerana*. In addition, chemosensory receptors and immune-related genes, which might be responsible for sophisticated and well-organized life styles in honey bees, were identified. Comparative studies of chemoreceptor gene families showed similarities in receptor family expansions relative to *A. mellifera*. However, significant differences in gene expression were also identified, potentially reflecting different capabilities for odor perception. In addition, immune-related genes in *A. cerana* exhibited expression patterns that may reflect an advanced social immune system, advancing understanding of the molecular basis of social immunity. This genome analysis will provide invaluable information on the novel characteristics of *A. cerana*, and contribute to the understanding of comparative sociogenomics with other sequenced social insect genomes.

## Methods

### Sample collection and DNA and RNA extraction

Adult drone bees of *A. cerana* (n = 4) were collected from a single colony at the College of Agriculture and Life Sciences, Seoul National University (SNU), Seoul, Korea during summer 2012. Mid-gut tissues were removed to reduce contamination with genomes of intestinal microorganisms. Genomic DNA was extracted from individual drone bees using the Wizard Genomic DNA Purification kit (Promega, MI, US) according to the manufacturer’s protocol. For RNA sequencing, *A. mellifera* and *A. cerana* worker bees were collected from 3 different colonies at SNU and stored immediately in liquid nitrogen. After flash freezing, samples were stored at −80°C until dissection. Tissues from *A. cerana* (brain, antenna, hypopharyngeal gland, gut, fat body, and venom gland, and larvae) and from *A. mellifera* (antenna) were dissected in cold RNase free PBS (pH = 7.4). Total RNA was isolated using a QIAGEN RNeasy Mini kit (Qiagen, CA, US) according to the manufacturer’s protocol.

### DNA and cDNA library construction

Genomic DNA for shotgun sequencing was sheared with average fragment sizes of 500 bp, 3 kb, and 10 kb. Three DNA libraries were constructed using the Illumina sequencing platform according to the manufacturer’s protocol (Illumina CA, US). Briefly, 5, 10, and 20 μg of genomic DNA were used for whole genome random shearing to construct 500 bp, 3 kb, and 10 kb libraries, respectively. Fragments were ligated with 3′ and 5′ adapters to produce libraries for PCR amplification.

For mRNA sequencing, cDNA libraries were prepared for each tissue using the Illumina mRNA sequencing kit (Illumina, CA, US) and the Clontech SMART cDNA Library Construction Kit (Invitrogen). Libraries were sequenced using the Illumina HiSeq2000 and Roche 454 Genome Sequencer FLX instrument (Roche, UK).

### *De novo*genome assembly and sequence analyses

Raw sequencing reads with phred scores ≤ 20 were filtered out using the CLC_quality_trim (CLC 3.22.55705). Duplicate sequences were removed with the remove_duplicate program (CLC-bio) using the default options. After filtration, genome libraries with inserts of 500 bp, 3 kb, and 10 kb were assembled using the AllPaths-LG (version 42411, [31]) algorithm with default parameters. The *A. cerana* genome sequence is available from the NCBI with project accession PRJNA235974. Repeat elements in the *A. cerana* genome were identified using RepeatModeler (version 1.0.7, [98]) with default options. Subsequently, RepeatMasker (version 4.03, [99]) was used to screen DNA sequences against RepBase (update 20130422, [100]), the repeat database, and mask all regions that matched known repetitive elements. Comparison of experimental mitochondrial DNA to published mitochondrial DNA (NCBI accession GQ162109) was performed using the CGView Server with the default options [101]. The percent identity shared between the *A. cerana* mitochondrial genome assembly and NCBI GQ162109 was determined by BLAST2 [102]. To examine the distribution of observed to expected (o/e) CpG ratios in protein coding sequences of *A. cerana*, we used in-house perl scripts to calculate normalized CpG o/e values [43]. Normalized CpG was calculated using the formula:


where freq(CpG) is the frequency of CpG, freq(C) is the frequency of C and freq(G) is the frequency of G observed in a CDS sequence.

### Evidence-based gene model prediction

Assembly of RNAseq data was performed using *de novo* assembler software, Trinity (version r2013-02-25, [98]). Alignment of RNAseq reads against genome assemblies was performed using Tophat and transcript assemblies were determined using Cufflinks (version 2.1.1, [103]). Gene set predictions were generated using GeneMark.hmm (version 2.5f, [99]). Homolog alignments were made using NCBI RefSeq and *A. mellifera* as a reference gene set (Amel_4.5). A final gene set was created synthetically by integrating evidence-based data using the gene modeling program, MAKER (version 2.26-beta), including the exonerate pipeline with default options [48, 104]. Subsequently, we performed blast searches with the NCBI non-redundant dataset to annotate combined gene models. All gene predictions were provided as input to the Apollo genome annotation editor (version 1.9.3, [105]), and genes included in phylogenetic analyses were manually checked against transcript information generated by Cufflinks to correct for 1) missing genes, 2) partial genes, and 3) separated genes.

### Gene orthology and ontology analysis

The protein sets of four insect species were obtained from *A. cerana* OGS v1.0, *A. mellifera* OGS v3.2 [32], *N. vitripennis* OGS v1.2 [36], and *D. melanogaster* r5.54 [106]. We used OrthoMCL v 2.0 [107] to perform ortholog analysis with default parameter for all steps in the program. GO annotation proceeded in Blast2GO (version 2.7) with default Blast2GO parameters. Enrichment analysis for statistical significance of GO annotation between two groups of annotated sequences was performed using Fisher’s Exact Test with default parameters.

### Gene family identification and phylogenetic analysis

Total 10,651 sequences of OGS v1.0 were classified with Gene Ontology (GO) and KEGG database using blast2GO (version 2.7) with MySQL DBMS (version 5.0.77). To search the sequence of *A. cerana* odorant receptors (Ors), gustatory receptors (Grs), and ionotropic receptors (Irs), we prepared three sets of query protein sequences: 1) first set includes Or and Gr protein sequences from *A. mellifera* (provided by Dr. Robertson H. M. at the University of Illinois, USA), 2) second set includes Or, Gr, and Ir protein sequences of previously known insects from NCBI Refseq [108], 3) third set includes functional domain of chemoreceptor from Pfam (PF02949, PF08395, PF00600) [109]. The TBLASTN of these three sets of receptor proteins was performed against *A. cerana* genome. Candidate chemoreceptor sequences from the results of TBLASTN were compared with *ab initio* gene predictions (see Gene annotation section) and verified its functional domain using the MOTIF search program [110]. Annotated Or, Gr, and Ir proteins were aligned with ClustalX [111] to corresponding proteins of *A. mellifera* and were manually corrected. Alignments were performed iteratively and each sequence was refined based on alignments to make complete Or, Gr, and Ir sequences for *A. cerana*. Sequences were aligned with ClustalX [111], and a tree was built with MEGA5 [112] using the maximum likelihood method. Bootstrap analysis was performed using 1000 replicates.

### Tissue expression analysis

To compare tissue transcriptomes of *A. cerana* and *A. mellifera*, RNAseq reads from antenna tissue of each species were mapped against annotated *Gr*, *Or*, and *Ir* gene sequences using RSEM [113]. TMM (Trimmed Mean of M-values)-normalized FPKM (fragments per kilobase per million) expression values were calculated by running perl scripts included in Trinity assembler (version r2013-02-25) and were used to draw a heat map [98].

### Analysis of microsynteny between AmOrs and AcOrs

Microsynteny analyses of orthologous *Or* genes between *A. cerana* and *A. mellifera* were carried out based on a phylogenetic tree. In addition, we performed reciprocal BLASTZ searches [114] between two species in syntenic regions and identified clear orthologs based on E-values (cut-off: 1e^−40^) and phylogenetic analyses.

### Immune related gene family

Holometabola immune gene orthologs obtained from Immunodb [96] were used as queries against the *A. cerana* genome using TBLASTN. Genomic sequences encoding immune gene candidates were compared with *ab initio* gene predictions to confirm completeness of the gene models.

### Availability of supporting data

The data sets supporting the results of this article are available through a BioProject (accession number PRJNA235974) at the NCBI.

### Data availability

The Illumina transcript data generated for *A. cerana* are available in the NCBI SRA [115] with accessions SRR1380970 (brain), SRR1380976 (antenna), SRR1380979 (hypopharyngeal gland), SRR1380984 (gut), SRR1388774 (fat body), SRR1406762 (venom gland), and the accession for 454 transcript data is SRR1408614 (head). The Illumina raw data from A*. mellifera* are available at the NCBI SRR1386316 (brain), SRR1407793 (hypopharyngeal gland), and SRR1408090 (antenna). Whole genome sequences of *A. cerana* are submitted in the NCBI Whole Genome Shotgun database (SUB582139). The genome and gene information is also available at *A. cerana* database (http://mnbldb.snu.ac.kr/genome_seq.php).

## Electronic supplementary material

Additional file 1: Table S1: Statistics of the honey bee genome assembly, *Apis mellifera* and *Apis cerana.* **Table S2.** Summary of repetitive elements in the *A. cerana* genome. **Table S3.** GO terms enriched in honey bee shared orthologs. **Table S4.** Immune related gene set counts in social and non-social insect, *Apis cerana, Apis mellifera, Nasonia vitripennis, Linepithema humile,* and *Drosophila melanogaster*. **Figure S1.** Syntenic view of the *A. mellifera* chromosome 3 and *A. cerana* scaffolds. **Figure S2.** Syntenic view of the mitochondrial genome of *A. mellifera* and *A. cerana*. **Figure S3.** The mitochondrial genome of *A. cerana*. **Figure S4.** Nomarlized CpG dinucleotide in protein-coding sequences in *A. cerana*. **Figure S5.** KEGG pathways in *A. cerana*. **Figure S6.** Phylogenetic tree of the gustatory receptor family. **Figure S7.** Phylogenetic tree of the odorant receptor family. **Figure S8.** Amino acid alignment between *AmOr11* and *AcOr30.* (PDF 2 MB)

Additional file 2: **Is a text file containing results of GO annotations for** ***A. cerana*** **OGS v 1.0.** (TXT 3 MB)

Additional file 3: **Is a spreadsheet showing results of KEGG analyses of** ***A. cerana*** **OGS v 1.0.** (XLSX 66 KB)

Additional file 4: **Is a spreadsheet showing results of GO enrichment analyses in** ***A. cerana*** **specific orthologs.** (XLSX 21 KB)

Additional file 5: **Is a spreadsheet showing results of reciprocal BLAST between** ***A. cerana*** **and** ***A. mellifera Or*** **genes.** (XLSX 17 KB)

## Abbreviations

Mya: Million years ago
SBV: Sacbrood virus
PE: Paired end
MP: Mate paired end
GC: Guanine plus cytosine
CpG: Cytosine followed by guanine in 5' to 3' orientation
TpG: Thymine followed by guanine in 5' to 3' orientation
o/e: observed/expected
DNMT: DNA methylation protein
TE: Transposable element
EST: Expressed sequence tag
RNA-seq: RNA sequencing
Refseq: Reference sequences
NCBI: National Center for Biotechnology Information
OGS: Official gene set
GO: Gene ontology
KEGG: Kyoto Encyclopedia of Genes and Genomes
Gr: Gustatory receptor
Or: Odorant receptor
Ir: Ionotropic receptor
9-ODA: 9-oxo-2-decenoic acid
iGluR: Ionotropic glutamate receptors
SBV: Sacbrood virus.
