# Supplementary material for: Uncovering the novel characteristics of Asian honey bee, Apis cerana, by whole genome sequencing
Source: BMC Genomics. 2015 Jan 2;16(1):1. doi: 10.1186/1471-2164-16-1 (PMC4326529; doi:10.1186/1471-2164-16-1)
Supplement: Supplementary file 1 — Additional file 1: Table S1: Statistics of the honey bee genome assembly, Apis mellifera and Apis cerana. Table S2. Summary of repetitive elements in the A. cerana genome. Table S3. GO terms enriched in honey bee shared orthologs. Table S4. Immune related gene set counts in social and non-social insect, Apis cerana, Apis mellifera, Nasonia vitripennis, Linepithema humile, and Drosophila melanogaster. Figure S1. Syntenic view of the A. mellifera chromosome 3 and A. cerana scaffolds. Figure S2. Syntenic view of the mitochondrial genome of A. mellifera and A. cerana. Figure S3. The mitochondrial genome of A. cerana. Figure S4. Nomarlized CpG dinucleotide in protein-coding sequences in A. cerana. Figure S5. KEGG pathways in A. cerana. Figure S6. Phylogenetic tree of the gustatory receptor family. Figure S7. Phylogenetic tree of the odorant receptor family. Figure S8. Amino acid alignment between AmOr11 and AcOr30. (PDF 2 MB) [file 12864_2014_6994_MOESM1_ESM.pdf]

Table S1. Statistics of the honey bee genome assembly, *Apis mellifera* and *Apis cerana*.

	**Amel_4.0** [a]	**Amel_4.5** [a]	**Acer_1.0**[b]
Total sequence length	231,029	229,734	228,316
Total assembly gap length	84,690	20,537	22,359
Number of scaffolds	10,742	5,644	2,430
Scaffold N50	359	997	1,422
Number of contigs	18,944	16,501	18,160
Contig N50	40	46	28
Total number of chromosomes	16	16	16 [1]

[a] Statistics information of Amel_4.0 and Amel_4.5 were obtained from [2].

[b] *A. cerana* genome assembly.

Table S2. Summary of repetitive elements in the *A. cerana* genome

Repeat type	Number of elements	Length occupied	Percentage of sequence
SINEs	0	0 bp	0.00 %
ALUs	0	0 bp	0.00 %
MIRs	0	0 bp	0.00 %
LINEs	0	0 bp	0.00 %
LINE1	0	0 bp	0.00 %
LINE2	0	0 bp	0.00 %
L3/CR1	0	0 bp	0.00 %
LTR elements	371	218,929 bp	0.10 %
ERVL	0	0 bp	0.00 %
ERVL-MaLRs	0	0 bp	0.00 %
ERV_class Ⅰ	0	0 bp	0.00 %
ERV_class Ⅱ	0	0 bp	0.00 %
DNA elements	924	247,574 bp	0.11 %
hAT-Charlie	0	0 bp	0.00 %
TcMar-Tigger	0	0 bp	0.00 %
Unclassified	19,307	3,982,110 bp	1.74 %
**Total interspersed repeats**		**4,448,613 bp**	**1.95 %**
Small RNA	0	0 bp	0.00 %
Satellites	0	0 bp	0.00 %
Simple repeats	183,134	8,167,274 bp	3.58 %
Low complexity	41,444	2,190,941 bp	0.96 %
**Total bases masked**		**14,794,603 bp**	**6.48 %**

Table S3.GO terms enriched in honey bee shared orthologs.

GO-ID	Term	Category	P-Value
GO:0007608	sensory perception of smell	P	1.75E-04
GO:0007606	sensory perception of chemical stimulus	P	7.55E-04
GO:0030536	larval feeding behavior	P	1.63E-03
GO:0001078	RNA polymerase II core promoter proximal region sequence-specific DNA binding transcription factor activity involved in negative regulation of transcription	F	2.41E-03
GO:0001227	RNA polymerase II transcription regulatory region sequence-specific DNA binding transcription factor activity involved in negative regulation of transcription	F	2.86E-03
GO:0015772	oligosaccharide transport	P	7.67E-03
GO:0008346	larval walking behavior	P	7.67E-03
GO:0018027	peptidyl-lysine dimethylation	P	7.69E-03
GO:0018023	peptidyl-lysine trimethylation	P	1.10E-02
GO:0051967	negative regulation of synaptic transmission, glutamatergic	P	1.17E-02
GO:0030537	larval behavior	P	1.35E-02
GO:0007631	feeding behavior	P	1.54E-02
GO:0008643	carbohydrate transport	P	1.58E-02
GO:1901476	carbohydrate transporter activity	F	1.87E-02
GO:0015144	carbohydrate transmembrane transporter activity	F	1.87E-02
GO:0051966	regulation of synaptic transmission, glutamatergic	P	2.11E-02
GO:0048172	regulation of short-term neuronal synaptic plasticity	P	2.21E-02
GO:0003680	AT DNA binding	F	2.24E-02
GO:0000982	RNA polymerase II core promoter proximal region sequence-specific DNA binding transcription factor activity	F	2.35E-02
GO:0038023	signaling receptor activity	F	2.37E-02
GO:0001071	nucleic acid binding transcription factor activity	F	2.52E-02
GO:0003700	sequence-specific DNA binding transcription factor activity	F	2.52E-02
GO:0043565	sequence-specific DNA binding	F	2.69E-02
GO:0000976	transcription regulatory region sequence-specific DNA binding	F	2.77E-02
GO:0016595	glutamate binding	F	2.88E-02

GO:0004888	transmembrane signaling receptor activity	F	3.04E-02
GO:0051119	sugar transmembrane transporter activity	F	3.16E-02
GO:0008066	glutamate receptor activity	F	3.19E-02
GO:0008345	larval locomotory behavior	P	3.92E-02
GO:0043388	positive regulation of DNA binding	P	3.97E-02
GO:0007196	adenylate cyclase-inhibiting G-protein coupled glutamate receptor signaling pathway	P	4.09E-02
GO:0070577	histone acetyl-lysine binding	F	4.09E-02
GO:0042800	histone methyltransferase activity (H3-K4 specific)	F	4.09E-02
GO:0048096	chromatin-mediated maintenance of transcription	P	4.09E-02
GO:0080182	histone H3-K4 trimethylation	P	4.09E-02
GO:0001012	RNA polymerase II regulatory region DNA binding	F	4.26E-02
GO:0061319	nephrocyte differentiation	P	4.29E-02

Table S4. Immune related gene set counts in social and non-social insect, *Apis cerana, Apis mellifera, Nasonia vitripennis, Linepithema humile,* and *Drosophila melanogaster*

Gene family	*A. cerana*	*A. mellifera*	*L. humile*	*N. vitripennis*	*D. melanogaster*
Recognition					
PGRP-S	1	3	4	3	7
PGRP-L	2	1	2	1	15
GNBP	2	1	4	3	7
Galectins	2	2	3	2	5
C-type lectins	5	10	NA	2	35
Scavenger receptor A	0	2	0	3	5
Scavenger receptor B	4	9	4	15	13
Scavenger receptor C	1	1	1	2	4
Signalling					
CLIP serine proteases	21	18	7	9	37
Serpin	1	3	4	6	30
Toll	3	5	9	9	9
Cactus	1	1	0	1	1
Dorsal	1	2	1	2	2
Relish	1	2	0	1	3
Effectors					
Prophenoloxidase	1	2	1	3	3
Defensins	2	2	1	2	1
Other immune peptides[a]	3	5	1	5	19
Lysozyme	2	3	2	3	14
TEP	3	4	3	1	6

[a]abaecin, apidaecin, hymenoptaecin,

Figure S1. Syntenic view of the *A. mellifera* chromosome 3 and *A. cerana* scaffolds.

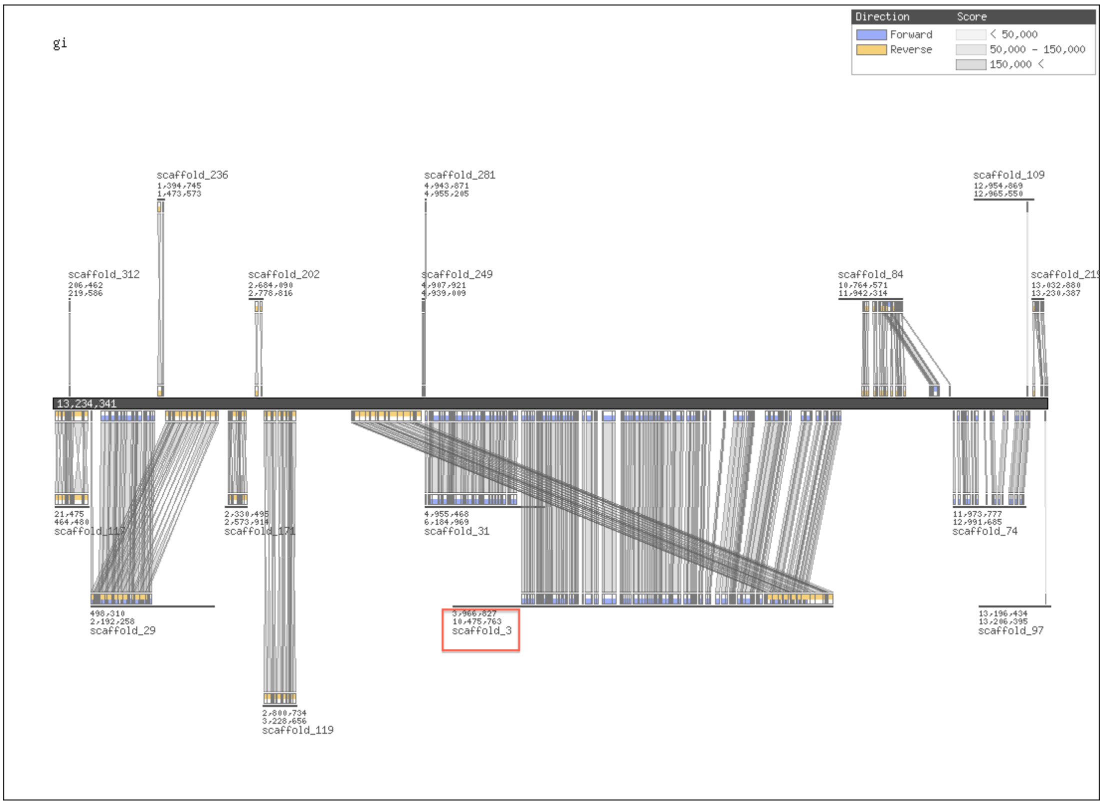

Figure S2. Syntenic view of the mitochondrial genome of *A. mellifera* and *A. cerana*.

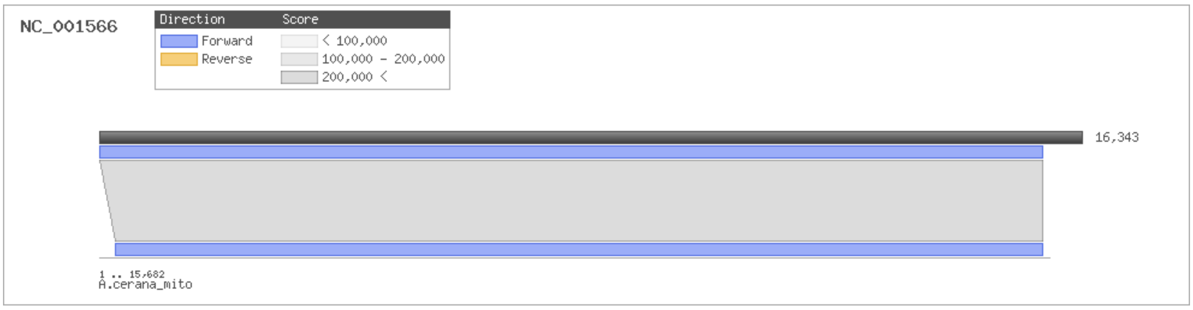

The pairwise syntenic region between the A. mellifera and A. cerana mitochondrial genomes conserved 99%.

Figure S3. The mitochondrial genome of *A. cerana*

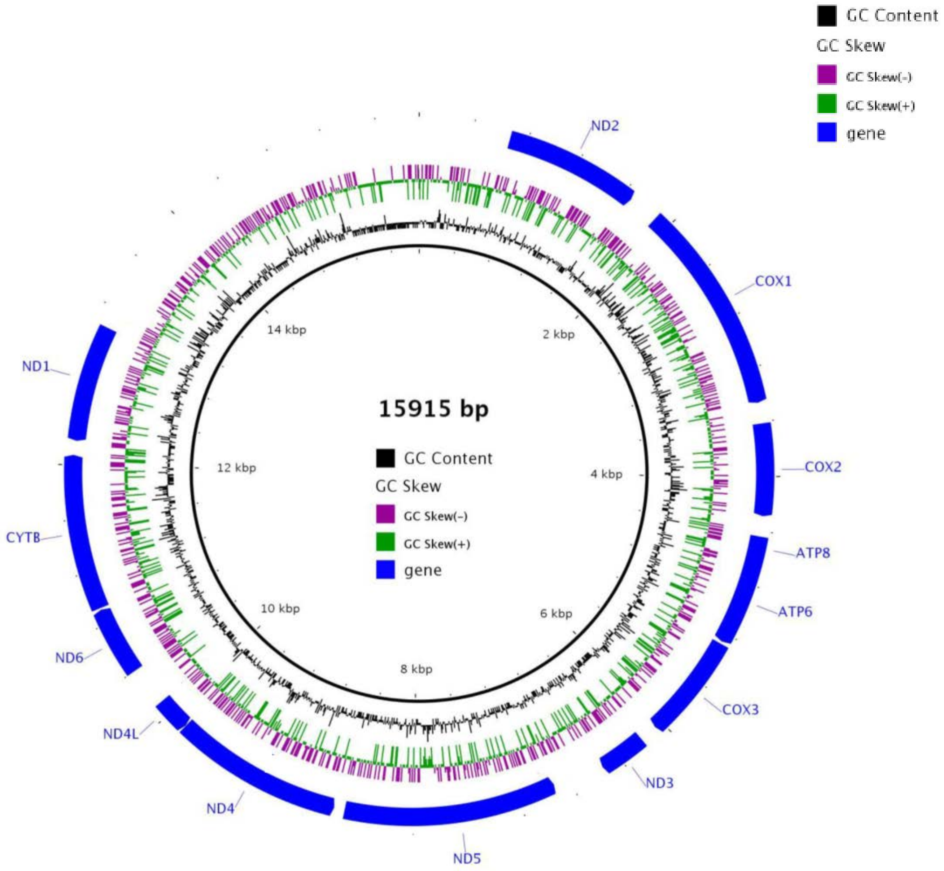

Figure S4. Nomarlized CpG dinucleotide in protein-coding sequences in *A. cerana*. X axis indicate normarlized dinucleotides observed/expected value and Y axis is CDS counts.

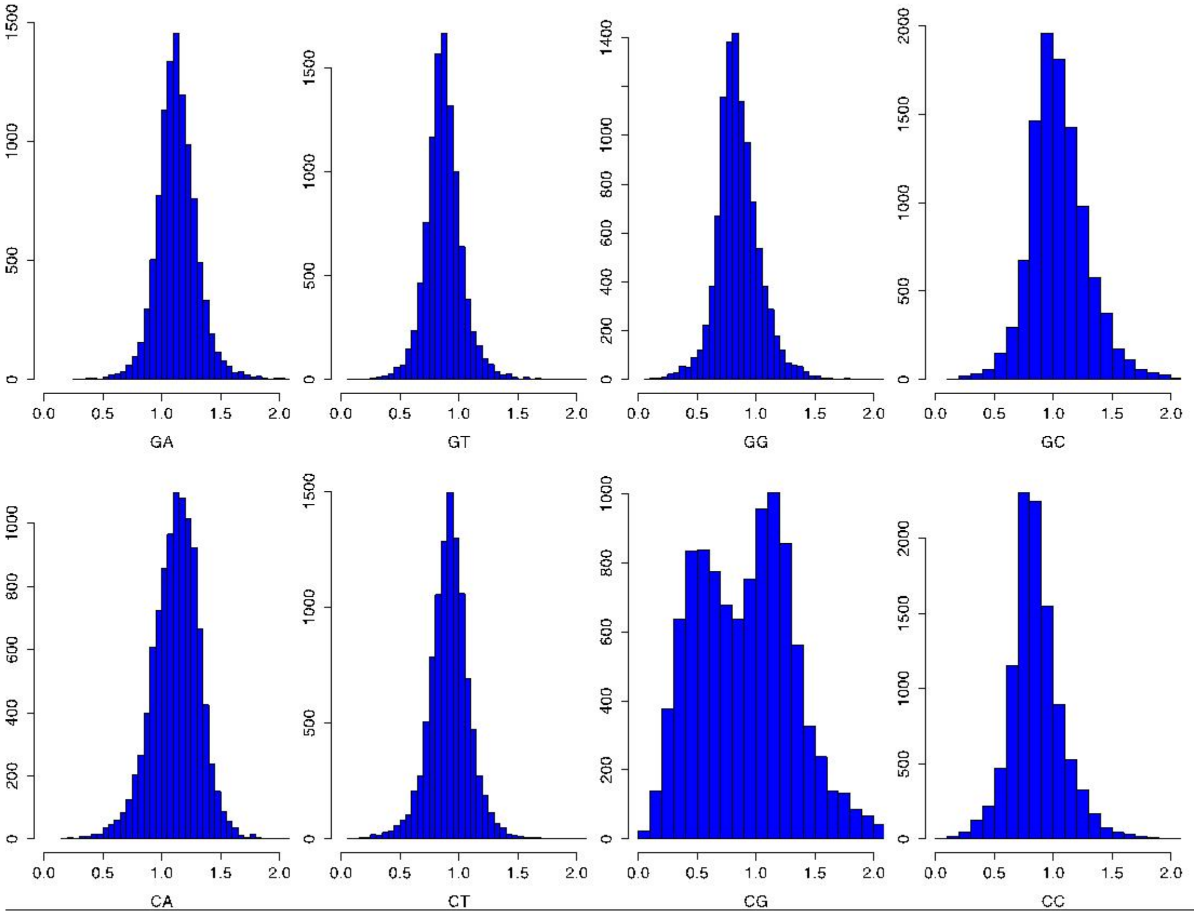

Figure S5. KEGG pathways in *A. cerana*. Genes with colored box identified against *A. cerana* OGS by using blast2go programs. (A) Fatty acid biosynthesis. (B) Glutathione metabolism. (C) Metabolism of xenobiotics by cytochrome P450.

(A) Fatty acid biosynthesis

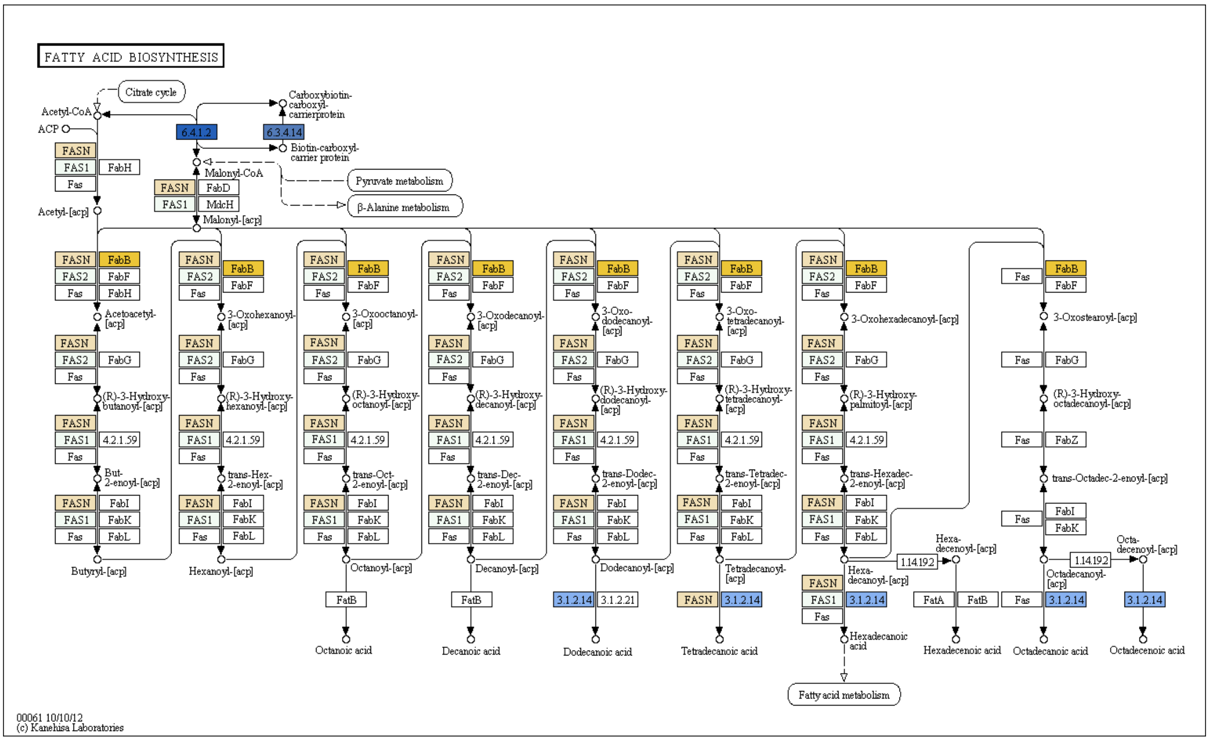

(B) Glutathione metabolism

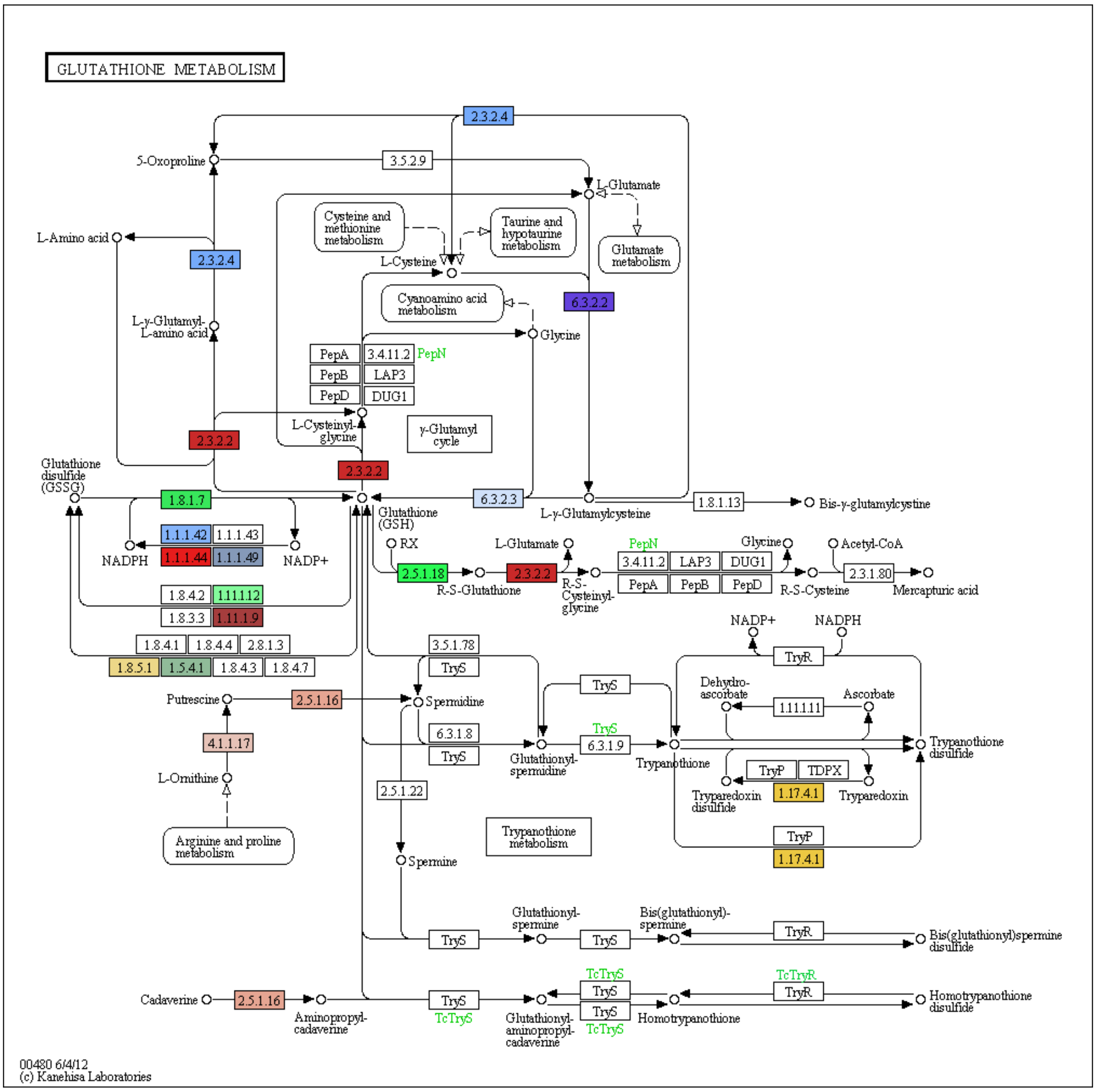

(C) Metabolism of xenobiotics by cytochrome P450

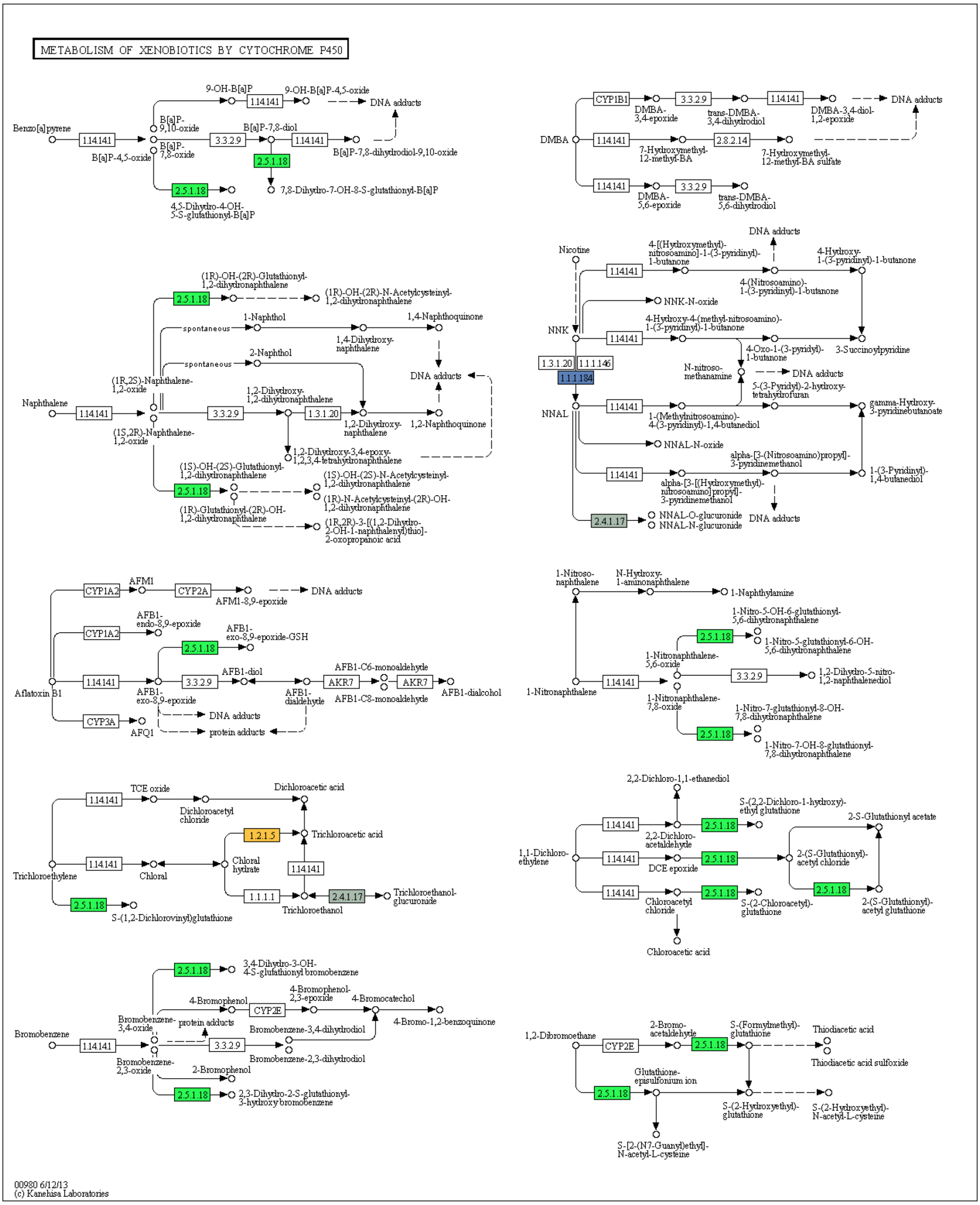

Figure S6. Phylogenetic tree of the gustatory receptor family Green, *D. melanogaster*; magenta, *A. mellifera*; blue, *N. vitripennis*; red, *A. cerana*

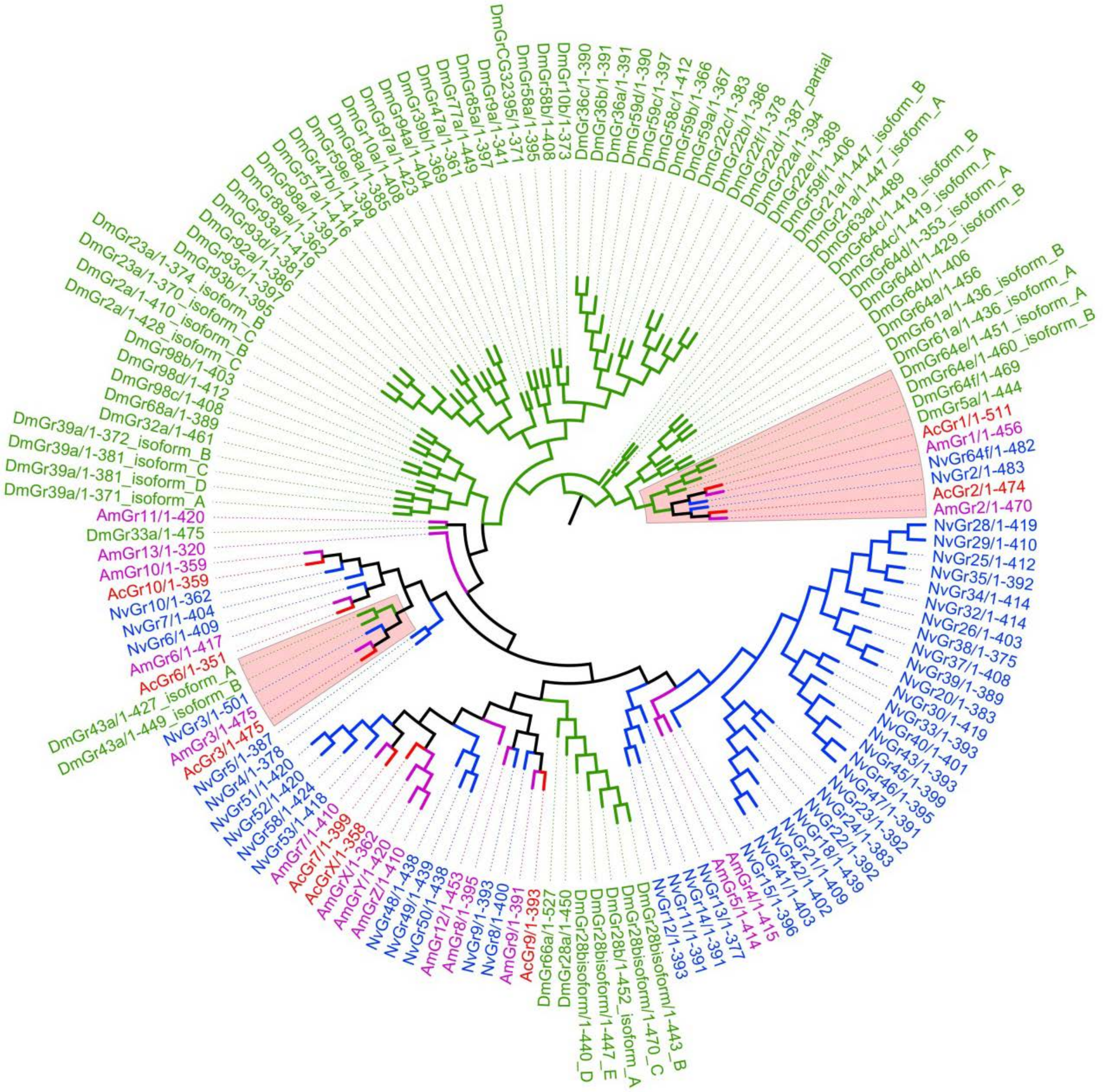

Figure S7. Phylogenetic tree of the odorant receptor family Green, *D. melanogaster*; blue, *A. mellifera*; orange, *N. vitripennis*; red, *A. cerana*

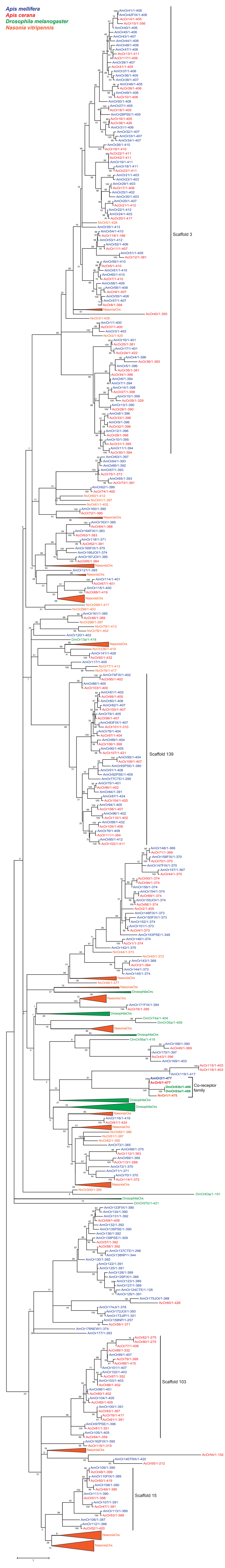

Figure S8. Amino acid alignment between *AmOr11* and *AcOr30*

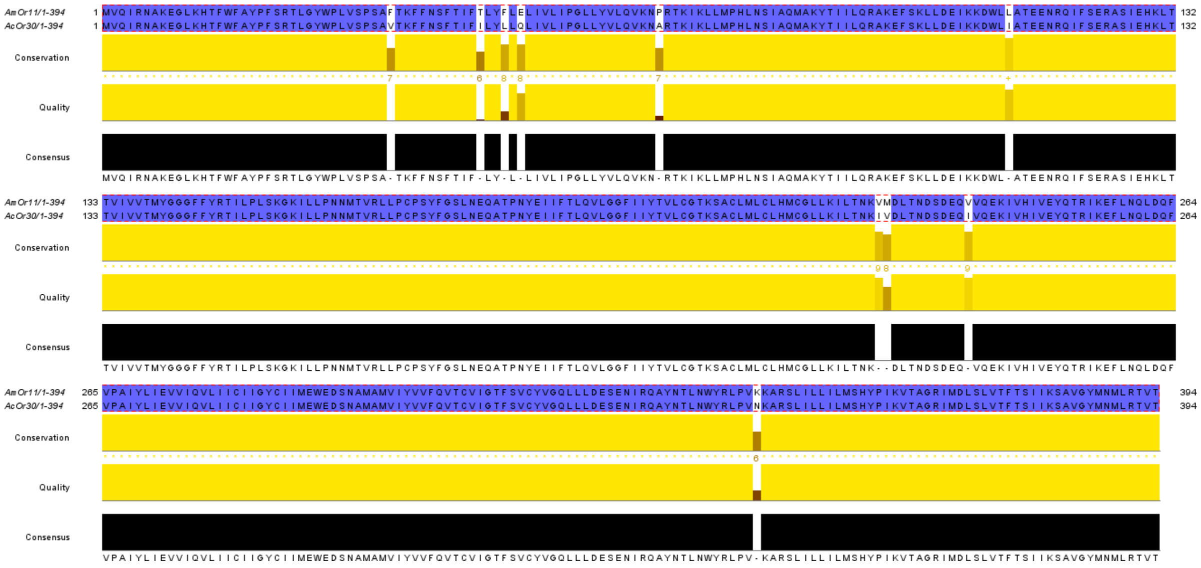

References

1. Shi YY, Sun LX, Huang ZY, Wu XB, Zhu YQ, Zheng HJ, Zeng ZJ: **A SNP based high-density linkage map of Apis cerana reveals a high recombination rate similar to Apis mellifera**. *PLoS One* 2013, **8**(10):e76459.
2. Elsik CG, Worley KC, Bennett AK, Beye M, Camara F, Childers CP, de Graaf DC, Debyser G, Deng J, Devreese B *et al*: **Finding the missing honey bee genes: lessons learned from a genome upgrade**. *BMC Genomics* 2014, **15**(1):86.
